# Health net-outcome objectives and approaches for spatial planning and development: a scoping review protocol

**DOI:** 10.11124/JBIES-23-00464

**Published:** 2024-06-25

**Authors:** James Stewart-Evans, Emma Wilson, Tessa Langley, Angela Hands, Jo Leonardi-Bee

**Affiliations:** 1 Nottingham Centre for Public Health and Epidemiology, University of Nottingham, Nottingham, UK; 2 Environmental Hazards and Emergencies Department, UK Health Security Agency, Nottingham, UK; 3 The Nottingham Centre for Evidence-Based Healthcare, University of Nottingham, Nottingham, UK; 4 Office for Health Improvement and Disparities, Department of Health and Social Care, UK

**Keywords:** health, net gain, no net loss, policy, spatial planning

## Abstract

**Objective:**

The objective of this scoping review is to review the body of knowledge on net gain and no net loss (net-outcome) objectives and approaches applicable to health in spatial planning and development policies and practice.

**Introduction:**

There is an established body of academic and gray literature addressing environmental net-outcome objectives, such as biodiversity net gain, in spatial planning policies and practice. A “health net gain” objective has recently been proposed as a driver for health protection and the realization of health. Such an objective and approach are yet to be scoped and defined.

**Inclusion criteria:**

This review will consider sources in the scientific and gray literature that describe health net-outcome objectives that can be implemented in spatial planning and development policies and practice. Source contexts will not be limited to specific countries, geographical areas, or settings. All types of evidence will be considered.

**Methods:**

This review will follow the JBI methodology for scoping reviews. Databases to be searched include PsycINFO, Embase, HMIC Health Management Information Consortium, MEDLINE (Ovid), Scopus, and selected databases from the ProQuest Social Science Premium Collection. Sources of gray literature to be searched include ProQuest Dissertations and Theses, TRIP Pro, and BASE. No language or date restrictions will be applied. Two independent reviewers will retrieve and review full-text studies and extract data. The results will be presented in tabular or diagrammatic format with a narrative summary.

**Review registration:**

Open Science Framework https://osf.io/4dbcm

## Introduction

Our built and natural environments are determinants of health.^1–4^ Health is addressed by national spatial planning policies as well as international and local requirements for the assessment of impacts associated with spatial plans and new development.^5–7^ While adverse impacts on nature and human health must typically be avoided or minimized, development can lead to damage despite the existence of protections.^1,8^ Conversely, spatial planning and development is recognized as a delivery mechanism for improvements to both the natural environment and human health.^4,9,10^ Environmental changes present potential opportunities to create health-promoting settings, address health-damaging risks, and reduce health inequalities.^4,11^ This encompasses neighborhood design and improvements to housing, recreational space, workplaces, public service settings, and transport environments, as well as the reduction of exposure to environmental hazards, such as air pollution, noise, and flood risk.

Sustainable development can be defined in spatial planning policy by broad economic, social, and environmental objectives across which net gains are sought.^5^ Environmental net-gain policy objectives set specific targets for measurable improvement of aspects of the natural environment.^12^ This can encompass stocks of natural capital and associated ecosystem services and flows of benefits, and reductions in environmental pressures such as pollution.^13–15^ Net-gain objectives can be implemented through spatial planning policy and practice, as evidenced by requirements in England’s spatial planning policy for new developments to deliver net gains in biodiversity (biodiversity net-gain).^5^ Associated development-level assessments delineate on- and off-site habitat losses and gains and their distribution and wider implications,^13,16^ and spatially defined environmental improvements provide place-based opportunities to address inequalities and improve social well-being.^17,18^

Practitioner and academic commentaries have suggested that policies and legislation with clear and explicit links to health may be needed to engage with developers and deliver improvements in healthy place-making.^19–21^ A “health net-gain” objective has recently been proposed as a driver for health protection and the realization of health improvement,^20,22,23^ but such an objective and approach for spatial planning and development policies and practices requires conceptual and methodological clarity and consideration of the practicalities and implications of implementation.

The phrase *health net gain* and its variants have found use in medical, public health, health economic, and other contexts in which changes in health are evaluated and health outcomes are described. As such, sources that describe assessments required by net-outcome policies can be found in many different domains. Health may be addressed by sources that consider:

- Ecological (nature-oriented) net outcomes: Such objectives are established in environmental and spatial planning policy and practice, and sources may describe characteristics or findings that are universally applicable to net-outcome policies. They may also explore co-benefits and the application of net-outcome objectives to broader social outcomes, including effects on people’s health and well-being.^13,17,18,24–26^
- Community or social outcomes: These outcomes address health as a distinct component or sub-objective.
- Regenerative design and development: This involves referencing concepts such as planetary health and addressing the health of human and natural systems in parallel or as an integrated whole.
- Specific determinants of health: These determinants address health in a narrower sense, such as the objective of delivering health net gains through changes in air quality associated with new developments.

Scoping reviews are useful when a body of literature is complex or heterogenous,^27^ as is the diverse academic and gray literature addressing net-outcome objectives and approaches that span domains and disciplines. Sources may describe generalized or universal net-outcome cases, as well as contexts more obviously related to spatial planning and development. Health may feature as a standalone (primary) objective or a secondary objective.

A preliminary search of PROSPERO, the Cochrane Database of Systematic Reviews (including health and social systems evidence), *JBI Evidence Synthesis*, EPPI Database of promoting health effectiveness reviews (DoPHER), Collaboration for Environmental Evidence Database of Evidence Reviews, Open Science Framework (OSF), PubMed, Scopus, Web of Science, and Figshare was conducted and no current or in-progress scoping or systematic reviews were identified that addressed health net gain or no net loss of health in the context of spatial planning policy objectives or approaches.

A scoping review of academic and gray literature is required to describe health net-outcome objectives and approaches for spatial planning and development policies and practices. The overarching objective of this scoping review is to scope the body of knowledge addressing net-outcome objectives and approaches applicable to health in spatial planning and development policies and practice. Related objectives are to clarify health net-outcome concepts and conceptual boundaries, describe how health net-outcome objectives are implemented in practice and the associated opportunities and challenges, and identify knowledge gaps in theory and practice.

The review is intended to inform future country-specific explorations of the potential characteristics and effects of a health net-gain objective and approach applicable to spatial planning policy and practice.

## Review questions

- What are the characteristics of health net-outcome policy objectives and the approaches that implement them in practice, including the rationales for their existence and use, and definitions of the objectives.
- How do health net-outcome policy objectives and approaches define health, and what are the principles or requirements that govern their implementation?
- What is the contextual positioning of heath net-outcome policy objectives and approaches and what are their effects or implications, including implementation opportunities and challenges?

## Inclusion criteria

### Concept

The overarching concept of interest to the review is health net-outcome objectives that can be implemented in spatial planning and development policies and practice. Sources will be included if they define, describe, or appraise health net-outcome objectives or approaches.

Health is primarily conceptualized as population health and well-being, but inclusion is not dependent on sources’ definitions of health, and sources directly referencing any form of health or well-being net-outcome objective will be included. Sources describing broader societal, social, and people-oriented net-outcome objectives encompassing health and well-being will be considered for inclusion. Sources describing health net-outcome objectives that address specific determinants of health or specific health outcomes will be included (eg, if a health net-gain objective is applied to activities that lead to changes in air quality or cardiorespiratory outcomes).

Sources describing other net-outcome objectives (such as environmental net gain) will be included if they describe the application of net-outcome objectives or elements of a net-outcome approach to health. Sources describing other net-outcome objectives (such as environmental net gain) that make no reference to health will be included only when their aim and main focus is the appraisal or conceptual elaboration of net-outcome-type policy objectives, or if they draw general principles from specific approaches.

Net-outcome *objectives* encompass goals, aims, targets, or requirements for, or principles of, no net loss or a net gain (or equivalently termed objectives). Sources describing *approaches* that implement net-outcome objectives in policies and practice will also be included. The review’s concept of a net-outcome approach will encompass sources that describe conceptual frameworks, theories, models, and definitions; underpinning ideological stances, implementation principles and their implications; and associated assessment and delivery requirements. Mitigation hierarchies are considered a feature of net-outcome approaches and sources describing them will be included. Sources that describe methodologies for the assessment of health costs and benefits will be excluded unless the methodology is explicitly linked to a net-outcome objective.

Sources that refer to health net outcomes in general terms without relating them to the core concept of a policy objective or approach (such as an evaluation reporting a net gain in health after an intervention) will be excluded.

### Context

Sources must describe objectives or approaches that are or can be implemented by, or are applicable to, spatial planning and development policies and practice.

*Implemented by* encompasses sources describing spatial planning principles; spatial planning and development planning policies; spatial planning practice guidance; spatial plans; design codes; development plans; and assessments of the effects of development projects, spatial plans, or planning policies.

The phrase *can be implemented* encompasses sources that do not explicitly address spatial planning and development but address place-making or the planning and governance of changes in natural and built environments related to land use, development, building, and infrastructure provision.

*Applicable to* encompasses sources that describe or evaluate the abstract concept of health net-outcome policy objectives or approaches without applying them to a particular domain. Sources that address health net-outcome objectives or approaches in other domains of policy or practice will only be considered if they either describe or evaluate the overarching concept (ie, of health net-outcome policy objectives or approaches) or make specific links to spatial planning and development planning policies and practice. Source contexts will not be limited to specific countries, geographical areas, or settings.

### Types of sources

Sources of evidence will include any type of evidence. The review will include sources from both scientific and gray literature.

## Methods

The proposed scoping review will be conducted in accordance with the JBI methodology for scoping reviews^28^ and be reported in line with the Preferred Reporting Items for Systematic Reviews and Meta-Analyses extension for Scoping Reviews (PRISMA-ScR).^29^

### Search strategy

The search strategy will not be limited by types of sources and aims to locate published and unpublished studies, reviews, opinion papers, policy and briefing papers, reports, articles, transcripts, and any other relevant material. It will target 3 domains: i) sources referencing the specific concept of health net gain; ii) sources referencing health terms and net-outcome concepts; and iii) sources that describe net-outcome concepts and features of policy objectives and approaches. To avoid inadvertent omission of sources describing net-outcome objectives in other transferable contexts, contextual relevance (to spatial planning and development) is to be determined during evidence selection.

An initial search of the online multidisciplinary databases Scopus and MEDLINE (Ovid) was conducted to identify articles that referenced synonyms of the phrase *health net gain* or the terms *health* and *net gain*. The text words contained in the titles and abstracts of relevant articles, and the index terms used to describe them, were reviewed to identify and incorporate additional phrases and terms related to the 3 domains described above and iteratively develop a full search strategy for MEDLINE (Ovid; See Appendix I). The search strategy will be replicated across all included information sources using, or adapting, all identified keywords and index terms. Simplified approaches will be used for gray literature databases with limited search functionalities. The reference lists of sources included in the review will be screened for additional sources.

To ensure a comprehensive review, articles published in any language from database inception to the present will be included. Translations from languages other than English will be sourced using DeepL (DeepL, Cologne, Germany) or via a translator.

The databases to be searched are PsycINFO, Embase, HMIC Health Management Information Consortium, MEDLINE (Ovid), International Bibliography of the Social Sciences (IBSS), Applied Social Sciences Index and Abstracts (ASSIA), PAIS Index, Political Science Database, Worldwide Political Science Abstracts, Social Science Database, Sociology Database, Social Services Abstracts, Sociological Abstracts, and Policy File (ProQuest), and Scopus.

Sources of gray literature to be searched include TRIP Pro, BASE, and ProQuest Dissertations and Theses (ProQuest). Limited additional web searches of Google will be conducted (using an adapted subset of journal database search strings and reviewing a maximum of 300 hits per search).

### Source of evidence selection

Search results will be imported into EndNote v.20.6 (Clarivate Analytics, PA, USA), de-duplicated, then transferred to Covidence (Veritas Health Innovation, Melbourne, Australia) for screening and data extraction, in line with the inclusion criteria.

A pilot test of a random sample of 20 titles and abstracts will be performed to refine eligibility criteria and reviewers’ guidance prior to the screening of studies. Next, the titles and abstracts of the first 10% of studies will be screened independently by 2 reviewers. If agreement is 90% or higher, the remaining titles and abstracts will be screened by 1 reviewer; otherwise, a further round of double screening will be conducted until a high level of agreement is reached (≥90%). All full-text articles of potentially eligible studies will be screened independently by 2 reviewers to determine eligibility. Any disagreements will be resolved by consensus or with a third reviewer. The reasons for exclusion of full texts will be reported in the full review. The results of the search will be presented in a PRISMA flow diagram.^29^

### Data extraction

A data extraction table (Appendix II) will be used to record key information from the sources and findings relevant to the review questions. Any further modifications will be detailed in the scoping review. Relevant text will be extracted verbatim using complete sentences or paragraphs for each item. Key information will be extracted from the first 10% of the selected sources independently by 2 reviewers. If agreement on charted data is 90% or higher, the remaining key information will be extracted by 1 reviewer; otherwise, a further round of double extraction will be conducted until a high level of agreement is reached (≥90%).

### Data analysis and presentation

Different types of health net-outcome objectives will be grouped under 2 overarching types of health net-outcome objective (health net gain and no net loss for health). If further subgroups or subcategories of health net-outcome objectives are found, findings may also be presented for distinct groups.

Descriptive content analysis of findings will be undertaken, including frequency counts of source characteristics (eg, objective[s] described, publication dates, and countries of application) and key findings (eg, definitions of health and implementation principles). Text excerpts with heterogenous findings will be summarized using alternative techniques for the visual representation of text data, such as word clouds.

A SWOT (strengths, weaknesses, opportunities, threats) analysis provides a simple tabular means of grouping and mapping contextual factors identified by a scoping review in a systematic manner whilst retaining specific details.^30^ It will be used to summarize extracted descriptions of positive or negative effects or implications of health net-outcome objectives and approaches (S and W), implementation opportunities (O), and implementation challenges (T). Diagrams may be used to illustrate relationships between specific characteristics of net-outcome objectives and approaches and SWOT factors.

Text may be recoded into keywords to facilitate data analysis, in which case the review appendices will report the original text associated with coded phrases or keywords. Any such coding will be basic and descriptive in nature.

Narrative summaries will describe overarching health net-outcome objectives, subcategories of objectives, and the range of findings for each. Results will be discussed in the context of current literature, practice, and policy. In particular, the review’s characterization of health net-outcome objectives and approaches and their implications will be compared with the literature describing environmental net-gain objectives and healthy planning and place-making principles. Unmet needs and gaps in existing research will be summarized, based primarily on charted data describing implementation challenges.

## Funding

JSE is the recipient of a PhD studentship funded by the UK Health Security Agency (UKHSA). Any views expressed in this protocol are those of the authors and not necessarily those of the UKHSA or the Department of Health and Social Care.

## Acknowledgments

Patricia Lacey, UKHSA knowledge and evidence specialist, and Alison Ashmore, University of Nottingham senior research librarian, for their assistance with the search strategy.

This review contributes towards a PhD for JSE.

## Author contributions

JSE and JLB conceived the idea. JSE developed the research protocol and methods, conducted searches, drafted and edited the manuscript, and led revisions in the revised manuscript. EW, TL, and AH helped to refine and develop the research protocol and methods and edited the final manuscript. JSE and AH piloted inclusion and exclusion criteria in Covidence. JLB provided methodological guidance, critical review, and editorial direction throughout the process. All authors approved the final manuscript submitted.

## Appendix I: Search strategy

### MEDLINE (Ovid)

Search conducted: August 17, 2023

**Table:**

Search	Query	Records retrieved
#1	(“net outcome*“ or “net gain*“ or “net positive outcome*“ or “net positive impact*“ or “no net loss*“ or “mitigation hierarch*“ or “regenerative design*“ or “regenerative development*“ or “regenerative sustainability”).mp.	1674
#2	(“net positive*“ or “net benefit*“ or “positive development*“).hw,kf.	166
#3	1 or 2	1839
#4	models, theoretical/ or concept formation/ or exp principle-based ethics/ or exp policy/ or policy making/ or exp social planning/ or professional practice/ or practice guideline/	460,455
#5	(theor* or concept* or principle* or policy or policies or plan or plans or planning or strategy or strategies or objective* or aim* or goal* or target* or requirement* or framework* or model* or approach* or practice*).hw,kf.	3,441,537
#6	health impact assessment/	945
#7	(“health risk assessment*“ or “impact assessment*“ or “environmental assessment*“ or “assessment tool*“ or “assessment method*“).mp.	67,702
#8	(HIA or HIAs or EIA or EIAs or “sustainability appraisal*“).mp.	11,932
#9	“risk evaluation and mitigation”/ or “compensation and redress”/	3212
#10	(mitigation* or “hierarchical system*“ or “response hierarch*“ or compensation* or offset* or “off-set*“ or tradeoff* or “trade-off*“ or reparation* or restitution*).mp.	193,777
#11	4 or 5 or 6 or 7 or 8 or 9 or 10	3,727,624
#12	((“net outcome*“ or “net gain*“ or “net benefit*“ or “net improve*“ or “net increase*“ or “net positive*“ or “positive outcome*“ or “net positive outcome*“ or “positive impact*“ or “net positive impact*“ or “positive development*“ or “regenerative design*“ or “regenerative development*“ or “regenerative sustainability” or “no net loss*“ or “mitigation hierarch*“) adj (theor* or concept* or principle* or polic* or plan* or strateg* or objective* or aim* or goal* or target* or requirement* or framework* or model* or assessment* or appraisal* or evaluation* or approach* or practice* or tool*)).mp.	123
#13	(3 and 11) or 12	581
#14	(health* or wellbeing or “well-being” or welfare).mp.	4,761,121
#15	exp health/ or exp public health/ or exp health status/ or exp health planning/ or health equity/ or exp social welfare/	9,618,315
#16	social problems/ or exp social justice/ or exp sociological factors/ or exp socioeconomic factors/	946,501
#17	(social* or societal* or society or societies or sociodemographic* or socioeconomic* or sociological*).hw,kf.	816,365
#18	humans/ or persons/ or exp population/ or population health management/	21,444,362
#19	(human* or population* or stakeholder* or community or communities).hw,kf.	21,645,926
#20	14 or 15 or 16 or 17 or 18 or 19	23,942,098
#21	3 and 20	1105
#22	((health* or wellbeing or “well-being” or welfare or soci* or commun*) adj3 net adj3 (gain* or benefit* or improve* or increase* or positive*)).mp.	675
#23	11 and 22	270
#24	(“health net gain*“ or “net health gain*“ or “wellbeing net gain*“ or “net wellbeing gain*“ or “well-being net gain*“ or “net well-being gain*“).mp.	25
#25	13 or 21 or 23 or 24	1647
No date or language limits applied		

## Appendix II: Draft data extraction instrument

**Table:**

Evidence source details and characteristics	
Source #	
Citation details	Author(s) Publication date Journal (or other publication source) Volume Issue Pages Sector (of source domain, if attributable)
Country of origin	
Type of evidence source (document type)	Journal article, gray literature, other
Document aims/purpose	(if applicable)
Health net-outcome objective, target, aim or goal	
Primary net-outcome objective, target, aim or goal	(for sources that present a health net-outcome objective as a secondary objective)
Country/countries of application	(the geographical context[s] the objective or approach applies to, if applicable)
**Details/results extracted from source of evidence**	
Health objective and approach: characteristics	
Rationale(s) for health net-outcome objective(s)	
Definition(s) of health net-outcome objective(s)	
Definition of “health” and/or health term(s)	(if given)
Implementation principle(s)	
Health objective and approach: contextual positioning and effects (SWOT)	
Positive effects or implications (Strengths)	
Negative effects or implications (Weaknesses)	
Implementation opportunities (Opportunities)	
Implementation challenges (Threats)	
